# Supplementary material for: Confounders mediate AI prediction of demographics in medical imaging
Source: NPJ Digit Med. 2022 Dec 22;5:188. doi: 10.1038/s41746-022-00720-8 (PMC9780355; doi:10.1038/s41746-022-00720-8)
Supplement: Supplementary file 2 — Supplemental Material [file 41746_2022_720_MOESM2_ESM.pdf]

Supplementary Table 1. Performance for models trained and evaluated on CSMC inpatient and outpatient echocardiograms stratified by location

	Sex Prediction [AUC]	Binary Race Prediction [AUC]	Age Prediction [MAE]
Inpatient	0.86 [0.85-0.86]	0.67 [0.65-0.68]	9.11 [8.92-9.30]
Outpatient	0.90 [0.89-0.91]	0.64 [0.61-0.67]	7.89 [7.63-8.15]
Overall	0.87 [0.86-0.88]	0.67 [0.66-0.68]	8.76 [8.60-8.91]

Supplementary Table 2. Race subgroup characteristics for Cedars-Sinai Medical Center

Apical 4 Chamber cohort

CSMC	White	Black or African American	Asian	Total
By Study				
Male n (%)	73,925 (58.5%)	14,791 (54.1%)	8,005 (55.1%)	96,721 (57.49%)
Female n (%)	52,468 (41.5%)	12,527 (45.9%)	6,536 (44.9%)	71,531 (42.51%)
Study Age mean (std)	67.68 (16.4)	63.0 (15.8)	65.1 (16.6)	66.69 (16.43)
Total	126,393	27,318	14,541	168,252
By Patient				
Male n (%)	11,036 (56.5%)	1,978 (48.7%)	1,140 (52.7%)	14,154 (54.99%)
Female n (%)	8,483 (43.5%)	2,080 (51.3%)	1,022 (47.3%)	11,585 (45.01%)
Total	19,519	4,058	2,162	25,739

Supplementary Table 3. Race subgroup characteristics for Stanford Healthcare cohort

**SHC**	**White**	**Black or African American**	**Asian**	**Total**
By Study				
Male n (%)	32,150 (57.04%)	2,490 (51.72%)	7,397 (52.17%)	42,037 (55,78%)
Female n (%)	24,217 (42.96%)	2,324 (48.28%)	6,781 (47.83%)	33,322 (44.22%)
Study Age mean (std)	61.85 (17.37)	56.21 (16.49)	60.07 (17.05)	61.15 (17.31%)
Total	56,367	14,178	4,814	75,359

Supplementary Table 4. Transducer models used in data collection

Transducer used for Image	White	Black or African American	Asian	Total
Philips Medical Systems: X5_1	89,120 (70.5%)	19,870 (72.7%)	10,398 (71.5%)	119,388 (71.0%)
Philips Medical Systems: S5_1	2,8484 (22.5%)	5,937 (21.7%)	3,281 (22.6%)	37,702 (22.4%)
Philips Medical Systems: X8_2t	4,130 (3.3%)	461 (1.7%)	390 (2.7%)	4,981 (3.0%)
Philips Medical Systems: X7_2t	1,116 (0.9%)	104 (0.4%)	96 (0.7%)	1,316 (0.8%)
Philips Medical Systems: S5-1	355 (0.3%)	103 (0.4%)	34 (0.2%)	492 (0.3%)
Philips Medical Systems: CX7_2t	223 (0.2%)	8 (0.0%)	18 (0.1%)	249 (0.1%)
Philips Medical Systems: S7_3t	24 (0.0%)	4 (0.0%)	0 (0.0%)	28 (0.0%)
Philips Medical Systems: S8_3	13 (0.0%)	6 (0.0%)	2 (0.0%)	21 (0.0%)
Philips Medical Systems: S5-2	6 (0.0%)	0 (0.0%)	5 (0.0%)	11 (0.0%)
Philips Medical Systems: S7_2omni	5 (0.0%)	4 (0.0%)	0 (0.0%)	9 (0.0%)
Philips Medical Systems: S2	8 (0.0%)	0 (0.0%)	0 (0.0%)	8 (0.0%)
Philips Medical Systems: S12_4	2 (0.0%)	3 (0.0%)	0 (0.0%)	5 (0.0%)
Philips Medical Systems: L12_3	5 (0.0%)	0 (0.0%)	0 (0.0%)	5 (0.0%)
Philips Medical Systems: X7-2T	2 (0.0%)	0 (0.0%)	0 (0.0%)	2 (0.0%)
Philips Medical Systems: C9_2	1 (0.0%)	0 (0.0%)	0 (0.0%)	1 (0.0%)
Philips Medical Systems: unknown	614 (0.5%)	130 (0.5%)	54 (0.4%)	798 (0.5%)
GE Vingmed Ultrasound: unknown	2,179 (1.7%)	683 (2.5%)	257 (1.8%)	3,119 (1.9%)
GE Healthcare Austria GmbH & Co OG: unknown	1 (0.0%)	0 (0.0%)	3 (0.0%)	4 (0.0%)
GE Healthcare: unknown	2 (0.0%)	0 (0.0%)	0 (0.0%)	2 (0.0%)
GE MEDICAL SYSTEMS: unknown	1 (0.0%)	0 (0.0%)	0 (0.0%)	1 (0.0%)
SIEMENS: V5M	47 (0.0%)	2 (0.0%)	2 (0.0%)	51 (0.0%)
SIEMENS: AcuNav10F	3 (0.0%)	0 (0.0%)	1 (0.0%)	4 (0.0%)
SIEMENS: 4V1c	0 (0.0%)	3 (0.0%)	0 (0.0%)	3 (0.0%)
SIEMENS: AcuNav8F	2 (0.0%)	0 (0.0%)	0 (0.0%)	2 (0.0%)
SIEMENS: Z6Ms	1 (0.0%)	0 (0.0%)	0 (0.0%)	1 (0.0%)
SIEMENS: unknown	2 (0.0%)	0 (0.0%)	0 (0.0%)	2 (0.0%)
Hitachi Aloka Medical,Ltd.: S12	4 (0.0%)	0 (0.0%)	0 (0.0%)	4 (0.0%)
MINDRAY: P4-2s	1 (0.0%)	0 (0.0%)	0 (0.0%)	1 (0.0%)
Unknown	42 (0.0%)	0 (0.0%)	0 (0.0%)	42 (0.0%)
Total	126,393 (100.0%)	27,318 (100.0%)	14,541 (100.0%)	168,252 (100.0%)
